# Are quality of life data reported in clinical trials evaluating CAR T cells in hematologic malignancies?

**DOI:** 10.1016/j.htct.2026.108542

**Published:** 2026-10-08

**Authors:** Julie Bouaziz-Bohbot, Lionel Karlin, Gilles A. Salles, Catherine Rioufol

**Affiliations:** a Lyon I University, EA 3738 - CICLY, Lyon Sud University, Lyon, France; b Hematology Department, Lyon Sud Hospital, Hospices Civils de Lyon, France; c Lymphoma Service, Department of Medicine, Memorial Sloan Kettering Cancer Center, NY, United States; d Clinical Oncology Pharmacy Unit, Lyon Sud Hospital, Hospices Civils de Lyon, France

**Keywords:** Car t-cells, Quality of life, Hematological malignancies, Clinical trials

## Abstract

**Background:**

Increasing indications for CAR T cells in early-line hematological malignancies underscore the need to report patient experiences. However, data regarding patient quality of life in this setting remain limited.

**Objective:**

This study aimed to assess the inclusion of Health-Related Quality of Life as an endpoint in clinical trials registered on ClinicalTrials.gov for CAR T-cell therapies in hematological malignancies.

**Methods:**

A comprehensive search of the U.S. National Institutes of Health database (ClinicalTrials.gov) was performed for trials registered up to August 28, 2022, using the keywords "CAR" AND ("lymphoma" OR "multiple myeloma" OR "leukemia" OR "hematologic malignancy"). For each selected trial, data regarding study design, setting, indication, treatment line, relevant outcomes, and evaluation scales were collected.

**Main results:**

Out of 1281 records, 429 trials met the inclusion criteria and were analyzed, with over half being phase 1. The most common primary endpoint was safety (47%). Health-Related Quality of Life was assessed as a secondary endpoint in only 11% of trials. A wide variety of questionnaires were identified, and data collection methodologies (baseline assessments, timepoints, and follow-up duration) varied considerably across studies.

**Conclusion:**

Establishing a standardized consensus for measuring Health-Related Quality of Life is essential to complement efficacy data in clinical trials and real-world studies evaluating CAR T-cell therapies.

## Introduction

Chimeric antigen receptor (CAR) T cells are an innovative and promising new class of treatments for patients with hematologic malignancies. This approach has already replaced standard of care (SOC) in early large B-cell recurrence, and continues to challenge immunochemotherapy, stem cell transplantation and even bispecific antibodies. This rapidly evolving landscape, in addition to health-economic considerations, raises the question of how to further evaluate these innovations.

Beyond assessing efficacy and tolerability, other aspects of health, including the patient’s experience, are now essential to appreciate the full impact of these therapies within a one-health perspective. Patient-reported outcome measures (PROMs), such as health-related quality of life (HRQoL), have become increasingly important for health authorities and clinicians in differentiating among treatment options [1]. Despite providing a better understanding of the patient's global status during treatment [2,3], there is surprisingly little HRQoL data in trials assessing CAR T cells in hematologic malignancy. Furthermore, the lack of a gold standard for assessing HRQoL leads to inconsistent methodology and assessment [4,5].

This study aimed to assess the role of HRQoL as an endpoint in trials registered on the US National Institutes of Health (NIH) ClinicalTrials.gov, evaluating the efficacy and safety of CAR T-cell therapy for hematologic malignancies. The results provide a basis for improving the design of future studies to better meet clinicians’ and health decision-makers’ need for knowledge.

## Methods

### Search strategy

A comprehensive search was performed of all clinical trials registered up to August 28th, 2022 in the US National Institutes of Health’s database registry, associating the keywords “CAR” and “lymphoma” or “multiple myeloma” or “leukemia” or “hematologic malignancy”. For all search results, a selection based on a series of criteria concerning study status, study phase and study type were added to refine the search. Then, the titles and abstracts were screened to verify that the studies aimed to evaluate the efficacy or safety of CAR T cells for hematologic malignancies.

### Clinical trial selection

Clinical trial eligibility was restricted to interventional studies evaluating CAR T cells, in any phase except early phase 1 or phase 4, at the following stages: recruitment (recruiting and not yet recruiting), active but not recruiting (the study is ongoing, but potential participants are not currently being recruited or enrolled), and completed (study ended normally, and participants are no longer being examined or treated). Studies that were terminated (stopped early), suspended or withdrawn, and studies enrolling by invitation or with an unknown status of recruitment were not eligible.

Trials evaluating CAR T cells in several hematologic malignancies were included only once.

Phase 1 or 2 clinical trials registered twice, once in phase 1 and once in phase 2, were identified as duplicates and removed. Other exclusion criteria were defined: studies not designed to assess the efficacy or safety of CAR T cells, studies assessing supportive drugs to prevent and to manage side-effects of CAR T cells, observational studies, and studies evaluating CAR-NK cells or genetically modified NK cells.

### Data extraction and analysis

The following items were collected in each selected trial: study design and setting, sample size, location of the trials, indication, line of treatment, objectives of the trial and criteria studied, relevant outcomes, HRQoL and scale if any. Two independent reviewers screened records and extracted data.

### Statistical analysis

Categorical variables were summarized as absolute numbers and percentages. Comparisons of proportions between groups were performed using the Pearson chi-square test of independence. When the sample was less than five, Fisher’s exact test was used instead. P-values <0.05 were considered statistically significant.

## Results

The registry search identified 1281 records for hematological malignancies. In total 429 relevant clinical trials were finally selected: 121 in lymphoma, 97 in leukemia, 80 in multiple myeloma, and 131 in two or more of these hematological diseases.

The category "two or more hematological malignancies" mainly concerned leukemia research trials.

### Clinical trial characteristics

The included studies were mostly conducted in Asia (China: n = 175; Japan: n = 17) and North America (US: n = 180; Canada: n = 24). Fifty-seven were conducted in Europe: Germany (33), Spain (30), Italy (29) and France (27). HRQoL was collected in 55.4% of trials promoted in Europe, 22.1% in North America, and 10.4% in Asia. The assessment of HRQoL in trials was significantly higher in Europe, with twice as many trials as in North America and five times as many as in Asia (χ2 = 80.62; p = 3.1 × 10^–18^). Table 1 presents the distribution according to phase. More than half were phase 1 trials (Table 1). The assessment of HRQoL in trials conducted in leukemia was significantly lower than in trials conducted in lymphoma and multiple myeloma (χ2 = 8.85; p = 0.03). HRQoL was rarely considered in Phase 1 trials compared to subsequent phases of development (p < 0.02).

**Table 1:** Distribution of the clinical trials assessing CAR T cells according to their phase (clinicaltrials.gov, August 28, 2022).

	Lymphoma - n	Leukemia - n	Multiple Myeloma - n	aSeveral hematological malignancies - n	Total of trials included - n (%)	Trials assessing HRQoL - n 9%)
Phase I	57	44	42	93	236 (55%)	9 (4%)
Phase I/II	31	35	24	35	125 (29%)	16 (13%)
Phase II	29	14	11	2	56 (13%)	15 (27%)
Phase II/III	0	3	0	0	3 (<1%)	0 (-)
Phase III	4	1	3	1	9 (2%)	6 (67%)
Total	121	97	80	131	429	46 (11%)
Trials assessing HRQoL – n (%)	18 (15%)	6 (6%)	13 (16%)	9 (7%)	46 (11%)	

HRQoL: Health-related Quality of Life.

a Lymphoma and/or multiple myeloma and/or leukemia.

### Trial status

More than half of the trials were in recruiting status, and nearly 17% were in active status, not recruiting. The remaining trials were in not yet recruiting (8%) or completed status (8%).

### Number of treated patients

As of August 28th, 2022, 17,254 patients with hematologic malignancies had been treated or registered in the US NIH library’s clinicaltrials.gov as intending to be treated with experimental CAR T cells. The 35 trials with completed status (i.e., studies that ended normally) enrolled 1341 patients.

The expected number of patients treated with CAR T cells in the 288 Recruiting status trials was estimated at 15,913 patients.

### Hematological malignancies and treatment lines

All clinical trials in lymphoma enrolled patients with relapsed or refractory (RR) disease, except for two that enrolled newly diagnosed patients. Over 73% of the trials in leukemia enrolled patients with RR acute lymphoblastic leukemia (ALL), with similar numbers of pediatric and adult trials. Seven of the 80 trials in multiple myeloma (MM) enrolled newly diagnosed patients, and four enrolled patients in second-line or later. >85% of the trials across multiple indications enrolled patients with RR B-cell hematological malignancies, including 36 studies in pediatric populations.

### Clinical trial endpoints

#### Survival

Overall survival was measured as a secondary endpoint in >60% of the trials.

The most common primary endpoint was safety, with 201 trials (47%) assessing the incidence of adverse effects and 111 trials (25%) measuring dose-limiting toxicity or maximum tolerated dose.

Ten other studies measured progression-free survival as the primary endpoint, and eight assessed complete response rate, or tumor load.

#### Health-related quality of life (HRQoL) and patient-reported outcomes (PRO)

HRQoL was assessed as a secondary outcome in 46 trials (11%), evaluating lymphoma (n = 18), multiple myeloma (n = 13), leukemia (n = 6), or multiple hematological malignancies (n = 9).

In total 11 different questionnaires and scales were used to collect HRQoL in these 46 trials (Table 2). The EQ-5D scale was used in half of the studies (23/46). In addition to generic scales such as the EORTC QLQ-C30 or the EQ-5D, some studies used specific questionnaires: FACT-Lym for lymphoma, QLQ-My20 for multiple myeloma or PedsQL for pediatric patients.

**Table 2:** Health-related Quality of life (HRQoL) scales used in clinical trials assessing CAR T cells in the treatment of hematological malignancies.

Hematologic malignancies	Trials assessing QOL – n (%)	Trials measuring quality of life using generic non-cancer specific scales (n)				Trials measuring quality of life using cancer specific scales (n)				
		EQ-5D-5L	NIH-PROMIS	SF-36	PGI-S	all cancer	pediatric cancer	specific cancer-disease		
						EORTC QLQ-C30	PedsQL	FACT-Lym	EORTC QLQMY20	MySIm-Q
		**Dimensions measured using the scale**								
		Medico-economic evaluation	Symptoms in all patients	Symptoms in all patients	Symptoms in all patients	QoL in all cancer patients	QoL in cancer pediatric patients	QoL in Lymphoma patients	QoL in Myeloma patients	QoL in Myeloma patients
		mobility, self-care, usual activities, pain and discomfort, anxiety and depression	physical, mental and social health	behavior, impairment, symptoms and social functioning	patient global impression of severity scale	Physical, role, cognitive, emotional functioning, general symptoms, global health status, QoL	Physical, emotional, social and school functioning	Physical, functional, emotional, social and family well-being, and lymphoma- related symptoms	Myeloma-related symptoms, side effects, emotional well being, and social functioning	Myeloma-related symptoms, side effects, body image, and future perspective
**Lymphoma**	18 (15%)	9	2	2	0	9	0	5	0	0
**Leukemia**	6 (6%)	3	1	0	0	2	1	0	0	0
**Multiple myeloma**	13 (16%)	8	0	0	4	10	0	0	8	3
a**Several hematological malignancies**	9 (7%)	3	0	0	0	6	1	0	0	0
**Total**	46 (11%)	23	3	2	4	27	2	5	8	3

a Lymphoma and/or multiple myeloma and/or leukemia (QLQ-C30: EORTC Core Quality of Life questionnaire [6]; EQ-5D-5L: EuroQol Group questionnaire including the visual analogue scale (VAS) [7]; NIH-PROMIS: NIH-Patient-Reported Outcomes Measurement Information System [8]; SF-36: 36-Item Short Form Survey [9]; FACT-Lym: Functional Assessment of Cancer Therapy - Lymphoma questionnaire [10]; PedsQL: Pediatric Quality of Life Inventory Measurement Model [11]; EORTC QLQ-MY20: EORTC Multiple Myeloma specific Quality of Life questionnaire [12]; MySlm-Q: Multiple Myeloma Symptom and Impact Questionnaire [13]; PGIS: Patient Global Impression of Symptom Severity scale [14]).

In 11 trials, HRQoL was collected at regular time points: baseline, month 1, month 3, then every 3 months until 6 (n = 2) or 12 (n = 7) months post-infusion. Two other trials reported that HRQoL was assessed at 1 and 6 months post-infusion. The time used as the baseline differed across the trials assessing HRQoL.

In other trials, the exact time of HRQoL collection was not specified, but the time period ranged from two years (n = 17) to five years (n = 6), and up to 15 years in some trials (n = 4). PROs as such were evaluated in four trials, all involving the indication of multiple myeloma.

## Discussion

This study is the first to report the use of HRQoL in clinical trials assessing CAR T cells in patients with hematologic malignancy registered in the US National Institute of Health's clinicaltrials.gov, with multiple endpoints analyzed.

CAR T cells have achieved unprecedented speed of access, after two phase-2 trials demonstrating their efficacy as third-line or more in patients with DLBCL [15,16] or ALL [17]. Their high cost per infusion [18] and health-economic impact, their positioning in increasingly early lines of treatment for more indications, and their challenging position in relation to both SOC and bispecific antibodies require strengthened evaluations, notably by introducing new endpoints such as HRQoL, and promoting real-life studies to supplement the results of clinical trials [19].

The present study highlights the dramatic inequality in access to CAR T cells worldwide, with >80% of trials conducted in the US and China. Only 11% of studies consider HRQoL as a secondary outcome, although this rate is higher than the 6.8% previously reported [20]. Documenting HRQoL and improving it through early supportive care could prevent treatment discontinuation [21]. Furthermore, this study is the first to reveal that the countries most actively developing CAR T-cell trials are those least interested in quality of life.

The present search found a wide variety of scales used to measure HRQoL, with a preponderance of the EORTC QLQ-C30 [6] and EQ-5D-5Lb [7]. The NIH PROMIS [8–22], which collects PROMs, and SF-36 [9] are also used to assess health status independently of pathology and treatment. The cancer-specific scales used in the clinicaltrials.gov trials are also consistent with the literature: Fact-Lym G [10] in lymphoma, QLQ-MY20 [12] in multiple myeloma, and PedsQL [11] for pediatric populations. Some authors use two scales for HRQoL measurement [4,5,23], although this raises issues regarding the burden imposed by the volume of data for patients and caregivers. The recent MDASI-CAR [24] scale, specific to CAR T cells, could be an interesting alternative; however, it does not allow comparison of CAR T cells versus other treatments such as SOC, stem-cell autograft, or bispecific antibodies.

Data are lacking on the duration of HRQoL collection and the time points. Duration of collection varied widely, from 2 to 15 years, with time points at baseline, month 1, month 3 and then every three months in most cases. In previous studies, data collection duration ranged from 90 days after treatment to five years [4,22,23,25]. The baseline is not standardized: prior to conditioning or salvage chemotherapy [4], patient eligibility screening [26], apheresis and/or before lymphodepletion [22], or renewed on the day of reinfusion [23]. Data collection timepoints included monthly assessments for the first 3 months post-infusion followed by quarterly evaluations [23], monthly assessments for 6 months followed by quarterly evaluations up to 24 months [24], or quarterly assessments up to 12 or 18 months post-infusion [26].

Various methods were used to assess HRQoL: self-administered paper questionnaires [23,27] by patients with or without the help of a caregiver (proxy) [23] or digital applications [5,28]; in some cases, methods were not specified [4]. Self-administered paper questionnaires incur a risk of data loss, non-completion and error when the data are re-entered electronically. Electronic collection seemed to be the most popular, encouraging the use of digital PROMs [28] but is less cost-accessible [27].

However, using an appropriate methodology, the results are highly significant, as shown by the pivotal ZUMA-7 [4], JULIET [26], and TRANSCEND [23] trials, with compliance rates of 80% and 90% in the multiple myeloma CARTITUDE-1 [29] and KARMMA [28] trials, respectively.

Compliance rates were lower in case of disease progression and decreased significantly with the length of follow-up [26].

HRQoL is essential for personalized management and can help clinicians choose between therapeutic strategies. In the pivotal randomized phase-3 ZUMA-7 study [4], axi-cel resulted in significant and clinically relevant improvement in HRQoL compared to SOC at days 100 and 150, faster recovery (by day 150 versus month 9), and less absenteeism and activity impairment at day 100, suggesting a faster return to baseline functioning in the workplace and at home. In the phase-I TRANSCEND [23] trial assessing lisocabtagene maraleucel (lisocel), an initial decline in HRQoL was observed during the first month for six months, followed by stabilization. In the phase-3 TRANSFORM study comparing lisocel versus SOC [5], patients in the lisocel arm showed clinically significantly better overall health and HRQoL.

HRQoL allows us to understand the experience of patients and to compare symptom burden and cognition reported after CAR T-cell treatment versus other strategies [30], and to investigate associations between perceived prognosis, quality of life and mood [31].

These findings call for consensus on HRQoL assessment methodology to increase the use of HRQoL as an endpoint in clinical trials and real-world studies. Guidelines are already available and can inform future developments. Chakraborty et al. provided recommendations for HRQoL collection time points tailored to the CAR T patient's care pathway: at baseline prior to lymphodepleting chemotherapy, at least weekly during the acute phase after CAR T-cell infusion, monthly until one year, and yearly thereafter [19]. Clinicians and scientific societies need to use these initial publications to harmonize methods for collecting and analyzing PROMs and HRQoL. Consensus is now crucial on the choice of HRQoL scale, collection duration and time points, definition of baseline, format, and the number of questionnaires to be completed by patients. The ethical question of whether to collect HRQoL data in patients whose condition is deteriorating will need to be clarified. HRQoL analysis should be integrated as standard practice in clinical trials and real-world studies. Finally, studies should be developed worldwide to promote equitable access to innovation.

## Limitations

This study is based on the ClinicalTrials.gov database. As HRQoL is not a primary endpoint in trials, there is a potential risk of incomplete data collection. Extracting and analysing the data with two independent reviewers should mitigate this risk. The database was last consulted on August 28th, 2022, and the consideration of quality of life in CAR T-cell trials may have evolved since then. It would be interesting to update this work to verify whether HRQoL is being considered more, and to establish whether geographical disparities persist. Notably, the countries that are most actively developing CAR T-cell trials are those with less emphasis on quality of life, which is an alarming finding.

## Conclusion

This study revealed a lack of consensus and heterogeneity in HRQoL assessment among patients treated with CAR T cells for hematologic malignancies. It is therefore crucial to establish a consensus on collecting HRQoL data in clinical trials and in real life, enabling all patients worldwide to benefit from these therapeutic innovations.

## Authorship

All authors have made substantial contributions to all of the following: (1) the conception and design of the study, acquisition of data, analysis and interpretation of data, (2) drafting the article or revising it critically for important intellectual content, (3) final approval of the version to be submitted.

## Conflicts of interest

The authors declare that they have no known competing financial interests or personal relationships that could have appeared to influence the work reported in this paper.
